# Factors affecting hospitalization and mortality in a retrospective study of elderly patients with heart failure

**DOI:** 10.1186/s12872-024-03871-6

**Published:** 2024-04-26

**Authors:** Johan Björklund, Louise Pettersson, Björn Agvall

**Affiliations:** 1 Hertig Knuts Vårdcentral, Region Halland, Halmstad, Sweden; 2 Department of Research and Development, Region Halland, Halmstad, Sweden; 3 Department of Clinical Sciences, Division of Pathology, Lund University, Lund, Sweden; 4 Center for Primary Health Care Research, Department of Clinical Sciences, Lund University, Malmö, Malmö, 202 13 Sweden

**Keywords:** Heart failure, Elderly, Risk factors, Hospitalization and mortality

## Abstract

**Background:**

Heart failure (HF) has a high prevalence in an elderly population and leads to a substantial hospitalization and mortality. The objective of this study was to investigate factors that affect hospitalization and mortality in an elderly population.

**Methods:**

A retrospective observational study was conducted of HF patients aged 76–95 years residing in Region Halland, Sweden. Between 2013 and 2019, a total of 3134 patients received a novel diagnosis of HF and were subsequently monitored for one year using data from a healthcare database. The patients were categorized into HF-phenotypes according to ejection fraction (EF) and those with HF diagnose solely based on clinical criteria with no defined EF. Cox regression analysis for hospital admissions and mortality was evaluated adjusted for pharmacotherapies, healthcare utilization and clinical characteristics.

**Results:**

Echocardiogram was performed in 56% of the patients and 51% were treated with recommended HF pharmacotherapy with betablockers combined with renin-angiotensin-aldosterone-system inhibition. The average number of inpatient days was 10.7 while the average number of visits to primary care physician was 5.4 and 8.7 to primary care nurse respectively. A Cox regression analysis for hospital admissions and mortality revealed that an eGFR < 30 ml/min was associated with a hazard ratio (HR) of 1.88 (confidence interval [CI] 1.56–2.28), elevated NT-proBNP with an HR of 2.09 (CI 1.59–2.76), diabetes with an HR of 1.31 (CI 1.13–1.52), and chronic obstructive pulmonary disease with an HR of 1.51 (CI 1.29–1.77). Having a primary care physician visit was associated to an HR of 0.16 (CI 0.14–0.19), and the use of recommended heart failure pharmacotherapy was associated with an HR of 0.52 (CI 0.44–0.61).

**Conclusions:**

In a Swedish elderly population with HF, factors such as advancing age, kidney dysfunction, elevated NT-proBNP levels, diabetes, and COPD were associated with an increased risk of both mortality and hospitalization. Conversely, patients who received recommended heart failure treatment and made regular visits to their primary care physician were associated with a decreased risk. This indicates that elderly patients with HF benefit from recommended HF treatment and highlights that follow-ups in primary care could be advantageous.

**Supplementary Information:**

The online version contains supplementary material available at 10.1186/s12872-024-03871-6.

## Introduction

Heart failure (HF) is a condition that affects approximately 2% of the population, with higher prevalence among the elderly population [1–3]. In individuals ≥ 80 years of age, it reaches around 11% for men and 14% for women [3]. Patients with HF of today have longer lifespans, and the general population is aging, which may explain the sustained or slightly increasing prevalence of HF observed over time [4–6]. Heart failure is not merely a singular pathological diagnosis; rather, it presents as a clinical syndrome characterized by cardinal symptoms such as breathlessness, ankle swelling, and fatigue, which may be accompanied by signs like elevated jugular venous pressure, pulmonary crackles, and peripheral oedema. This syndrome arises from structural and/or functional abnormalities of the heart, leading to elevated intracardiac pressures and/or inadequate cardiac output both at rest and during physical exertion [7]. This chronic condition often leads to significant distress for those affected and the mortality rate is high. The healthcare utilization is high for patients with HF and results in substantial societal costs, primarily due to a frequent need of hospitalization [8–12]. The HF-related one-year readmission rate may be as high as 38% [13].

Measuring the ejection fraction (EF) preferably with echocardiography is important in order to assess the cardiac function and determine the HF phenotype. The different phenotypes of HF also have different recommended pharmacological treatment [7, 14]. Based on EF, HF is subcategorized into three phenotypes: HF with reduced EF (HFrEF), HF with mildly reduced EF (HFmrEF) and HF with preserved EF (HFpEF). In accordance with guidelines, the pharmacotherapy recommended for HF has demonstrated a reduction in mortality, healthcare utilization, and an improvement in quality of life (QoL) [7, 14–16]. However, the pharmacotherapy for HFpEF has previously been uncertain, but recent guidelines recommend treatment with SGLT-2 inhibitors. Furthermore, it is recommended to employ diuretics for fluid retention management, along with addressing any comorbidities that could be contributing to the condition [7, 14, 17]. It has been established that the use of recommended pharmacotherapy for HFrEF is similarly effective in the elderly as in the younger patients [18]. As a result, it is crucial to accurately identify the specific phenotype of HF in both younger and elderly patients when deciding on the most appropriate treatment approach.

Elderly people with HF are frequently visiting primary healthcare centres and a previous study showed that high age is strongly correlated with lower likelihood of follow-up in specialty care [19]. Previous studies conducted in Region Halland (RH), Sweden, have focused on an incident HF cohort. In this cohort, 57% were examined with echocardiography to establish the HF phenotype [20]. In a follow-up study based on the same cohort, patients admitted to hospital with a diagnosis of HF were followed for 100 days after discharge [21]. Of these patients 73% were > 75 years of age and both studies revealed a significant risk of mortality and hospitalization for this group of patients.

This has raised an interest in understanding how older patients are managed and what factors contribute to the risk of mortality and the influence of hospitalization in this age group. The objective of this study was to investigate important factors in the assessment of elderly patients regarding risk of hospitalization and mortality in a Swedish healthcare setting.

## Methods

A retrospective population-based study was conducted of a cohort including HF individuals > 75 years of age with HF in Region Halland (RH), located in southwestern Sweden, which has an approximate population of 330,000 residents. Most healthcare services offered to the residents are funded by the regional government, which provides inpatient care, specialized outpatient care, and primary healthcare. RH maintains and operates two acute care hospitals and one elective hospital in the area. There are 48 primary care clinics in RH, of which approximately half are privately managed but funded by the region.

### Data sources

The data information was retrieved from the Regional Healthcare Information Platform (RHIP) [21]. This system contains pseudonymized data on all healthcare, visits and utilization in the area, gathered routinely during standard care. The information collected from primary and hospital care, laboratory tests, and echocardiogram was used for this research. Pharmacological data of treatment was gathered from the Swedish Prescribed Drugs Register and the pharmacy’s dose dispensing system (Apodos) through RHIP.

### Study population

**Fig. 1:**
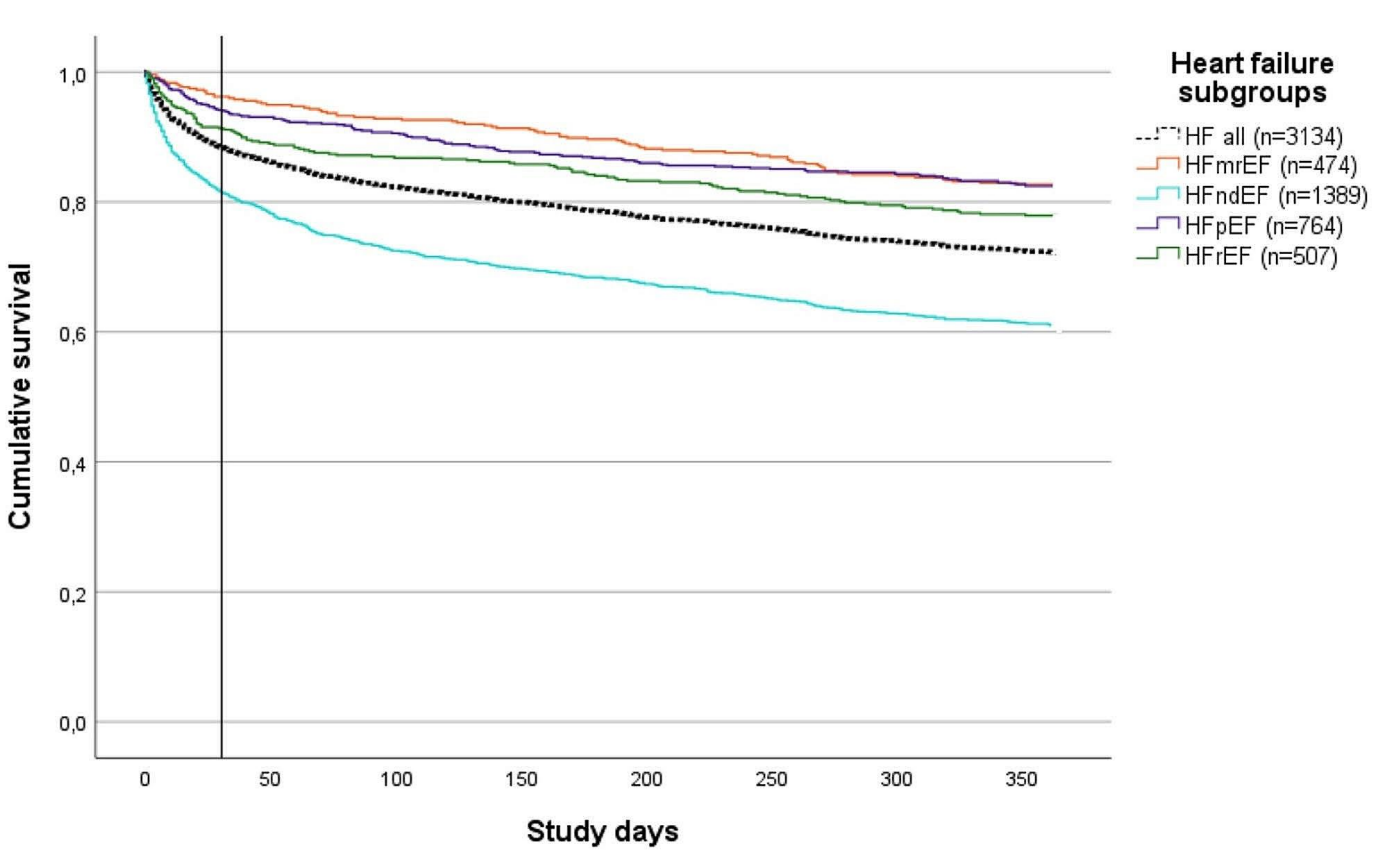
The all-cause mortality during the study period within one year

The study included individuals aged > 75 years when first-time diagnosed with HF according to ICD codes I110, I420, I423 – I432, I438, I500 – I501, I509. The diagnosis was documented by a physician’s journal entry between 2013 and 2019 in RH according to ICD-10. Only patients who received their healthcare and were residents of RH during the follow-up period were considered. The study excluded patients ≤ 75 years of age, > 95 years of age when first diagnosed with HF, and those patients with no healthcare encounters within one year after their diagnosis. Individuals living in other regions of Sweden or visitors from abroad seeking temporary medical care in RH were also excluded from the analysis. In total, there were 3134 patients between 76 and 95 years diagnosed with HF during the study period. The patients were observed for one year from onset of their HF diagnosis. A flow diagram showing the study’s methodology can be found in Appendix - Fig. 1.

### Study procedure

For the study population, age, sex and comorbidities (displayed in Table 1) were recorded. Levels of N-terminal pro–B-type natriuretic peptide (NT-proBNP) were collected, as were estimated glomerular filtration rate (eGFR).

**Table 1:** Basic characteristics regarding gender, age, comorbidities, renal function and natriuretic peptides

	HFrEF	HFmrEF	HFpEF	HFndEF	Total	P-value
Total, n (%)	507 (16)	474 (15)	764 (24)	1389 (44)	3134 (100)	
*Gender*						
Women, n (%)^1^	197 (42)	765 (55)	420 (55)	187 (37)	1569 (50)	< 0.001^1^
*Age groups*						
76–85 years, n (%)	329 (65)	302 (64)	463 (61)	615 (44)	1709 (55)	< 0.001^1^
86–95 years, n (%)	178 (35)	172 (36)	301 (39)	774 (56)	1425 (46)	
Age years, mean (SD)^2^	83.4 (5.2)	83.5 (4.9)	84.0 (5.0)	86.1 (5.1)	84.7 (5.2)	< 0.001^2^
*Comorbidities*						
Hypertension, n (%)	348 (69)	371 (78)	623 (82)	1073 (77)	2415 (77)	0.22^1^
IHD, n (%)	310 (61)	252 (53)	258 (34)	502 (36)	1322 (42)	< 0.001^1^
CVI, n (%)	77 (15)	86 (18)	126 (17)	284 (20)	573 (18)	0.002^1^
Atrial fibrillation, n (%)	259 (51)	284 (60)	416 (55)	696 (50)	1655 (53)	0.002^1^
Diabetes, n (%)	118 (23)	100 (21)	162 (21	300 (22)	680 (22)	0.81^1^
COPD, n (%)	61 (12)	71 (15)	114 (15)	207 (15)	453 (15)	0.42^1^
*Origin of diagnosis*						
In-patient care	353 (70)	266 (56)	354 (46)	698 (50)	1671 (53)	< 0.001^1^
Out-patient care	60 (12)	66 (14)	57 (8)	41 (3)	224 (7)	
Emergency department	24 (5)	31 (7)	56 (7)	88 (6)	199 (6)	
Primary care	70 (14)	111 (23)	297 (39)	562 (41)	1040 (33)	
*Kidney function*						
eGFR ml/min, mean (SD)	49.5 (16.4)	49.7 (15.5)	50.1 (16.1)	48.6 (17.2)	49.4 (16.6)	0.08^2^
> 60 ml/min, n (%)	142 (28)	128 (27)	248 (33)	402 (29)	920 (29)	0.03^1^
30–60 ml/min, n (%)	298 (59)	295 (62)	427 (56)	778 (56)	1798 (57)	
< 30 ml/min, n (%)	67 (13)	51 (11)	89 (12)	209 (15)	416 (13)	
eGFR missing	0	0	0	0	0	
*NT-proBNP levels*						
NT-proBNP ng/l, mean (SD)	9135 (11,697)	4957 (7438)	3715 (6105)	5051 (9271)	5397 (8968)	< 0.001^2^
HF unlikely, n (%)	9 (2)	24 (5)	56 (7)	127 (9)	216 (7)	< 0.001^1^
Grey zone, n (%)	101 (20)	142 (30)	287 (38)	423 (31)	953 (30)	
HF likely, n (%)	370 (73)	274 (58)	375 (49)	577 (42)	1596 (51)	
Missing, n (%)	27 (5)	34 (7)	46 (6)	262(19)	369 (12)	

*Note* HFrEF = heart failure with reduced ejection fraction, HFmrEF = heart failure with mildly reduced ejection fraction, HFpEF = heart failure with preserved ejection fraction, HFndEF = heart failure with no defined ejection fraction, n = number, SD = standard deviation, IHD = Ischemic heart disease, CVI = cerebrovascular insult, COPD = chronic obstructive pulmonary disease, eGFR = estimated glomerular filtration rate (ml/min), NT-proBNP = natriuretic terminal pro brain natriuretic peptide, HF = Heart failure

^1^ Pearson Chi-Square

^2^ One-way ANOVA

The measurement of cardiac function relied on the assessment of EF obtained from echocardiograms, and this assessment was accomplished using an artificial intelligence technology that accessed data from digital medical records [20–22]. The echocardiogram data enabled the classification of the patients into three HF phenotypes based on EF measurements: HFrEF for EF < 40%, HFmrEF for EF between 40 and 49%, and HFpEF for EF ≥ 50%. Patients diagnosed with HF but lacking records of a diagnostic echocardiogram, with diagnoses solely based on clinical criteria, were categorized as HF with no defined EF (HFndEF). Variability among examiners in measuring ejection fraction (EF) during echocardiography is a potential concern. These individual differences have not been accounted for and the data relied solely on the documentation available in the electronic medical records.

The NT-proBNP measurements obtained were at the time of the first HF diagnosis at index, within the period three months before and after index. When several measurements, the highest value was registered as it was considered the most representative for the patient’s cardiac function. These NT-proBNP levels were categorized into three groups, representing the likelihood of association with HF. Patients with normal NT-proBNP levels were classified as “HF unlikely,” while those with elevated NT-proBNP levels fell into either the “grey zone” or “HF likely” categories [23–26]. NT-proBNP levels < 300 pg/ml were defined as “HF unlikely”. “Grey zone” for HF was defined when a patient had NT-proBNP levels at 300–1800 pg/ml. “HF likely” was defined as patients with an NT-proBNP level > 1800 pg/ml.

Kidney function was estimated from the eGFR (ml/min/1.73m^2^) value, that was within three months before and after and closest to the time of diagnosis [27]. Kidney function was classified as normal with an eGFR ≥ 60 ml/min, lowered with an eGFR 30-59 ml/min or impaired with an eGFR < 30 ml/min.

All laboratory samples were analysed following the same procedure and within the same clinic.

The pharmaceuticals used for the treatment of HF were beta-blockers, renin-angiotensin-aldosterone system inhibitors (RAASi), mineralocorticoid receptor antagonists (MRA), and diuretics which are specified in detail in Appendix-Table 2 [7, 14]. Patients who during the first year after diagnosis had three or more medication pickups of each type of pharmaceutical from the pharmacy, were considered to receive pharmacological treatment of the corresponding type. Those patients that were alive within the 0–90 days and 91–180 days periods from the index date were classified as having adherence treatment if they had a minimum of one pharmacy pickup for each prescribed medication or two or more pickups, respectively [10]. Otherwise, it was assessed that they did not continuously take the medication. At the time of this study, the recommended first-line treatment for all patients with HF was beta-blockers and RAASi. According to the 2021 European guidelines, MRA, Angiotensin receptor neprilysin inhibitors (ARNI), and Sodium glucose cotransporter-2 antagonists have also been included as first-line treatment options. Pharmacological treatment was divided into beta-blockers, RAASi, MRA and diuretics separately as well as beta-blockers and RAASi in combination and triple therapy with beta-blockers, RAASi and MRA. ARNI was identified in 33 patients and is thus categorized as a RAASi since ARNI contains valsartan.

The utilization of healthcare services was obtained and categorized into inpatient and outpatient care. Inpatient care pertains to the quantity of hospital admissions and the length of hospital stays, irrespective of the cause. The number of visits to the emergency department by patients in RH was recorded. Outpatient care includes visits to healthcare professionals such as physicians, nurses, and paramedical staff (including curator/psychologist, physiotherapist, occupational therapist) for hospital-based treatment. In primary care, the number of visits to doctors, nurses, and paramedical staff was documented.

### Statistical analysis

The basic of characteristics is presented with descriptive statistics. Pearson Chi-Square tests were used when analysing categorical data as well as one-year. Continuous variables and mean values were analysed using one-way ANOVA when comparing several groups.

Kidney dysfunction is recognized for its ability to increase NT-proBNP levels, and a correlation analysis was conducted to ascertain whether kidney dysfunction could be a potential factor contributing to erroneously elevated NT-proBNP levels [28].

To display the all-cause mortality over time, a Kaplan-Meier plot was analysed for the total study cohort and separated for each HF subgroup (HFrEF, HFmrEF, HFpEF and HFndEF).

A Cox regression analysis was conducted to assess hospital admission, as the dependent variable, while adjusted for various factors. These factors included HF-subgroup, categorized kidney function and NT-proBNP levels, the use of beta blockers in combination with RAASi, and primary care visits to physician. The analysis took into account the time each patient lived throughout the study period.

Two Cox regression analyses were performed with the outcome of more than one hospital admission, and one-year all-cause mortality within the study period. Both Cox regression analyses were adjusted for HF subgroups, kidney function, NT-proBNP levels, comorbidities, pharmacotherapy, and visits to primary care physicians.

A p-value < 0.05 was considered significant. The data was analysed with the computer program IBM SPSS Statistics version 29.

### Ethical considerations

The research conducted in this study received approval from the Swedish Ethical Review Board, Stockholm Department 2 Medicine, registration number 2020 − 00455. Given the retrospective nature of the study, the requirement for informed consent was waived, and the study procedures obtained approval from the Swedish Ethical Review Board. All methods and procedures undertaken in this study adhered to applicable guidelines and regulations.

## Results

There were 3134 patients who fulfilled the inclusion criteria and an echocardiogram appeared in 1745 (56%) patients. Among these, 507 (16%) were classified as HFrEF, 474 (15%) as HFmrEF, and 764 (24%) as HFpEF. The remaining 1389 (44%) patients who had no echocardiogram were categorized as part of the HFndEF group. The study population consisted of 1425 (46%) patients ≥ 85 years of age, divided into 1569 (50%) women and 1565 (50%) men (Table 1). Results regarding comorbidities, kidney function, NT-proBNP levels and origin of diagnosis, in total and in HF subgroups, are shown in Table 1. There were more women in the HFpEF and HFmrEF groups, and more men in the HFrEF and HFndEF groups. It was 56% of the patients that had an echocardiogram. Within the age group of 85–95 years, 46% had undergone echocardiogram, contrasting with the 64% rate among patients aged 76 to 84 years. There were 8% of the patients having a HF diagnosis based on clinical criteria, without an echocardiogram nor NT-proBNP.

The distribution of pharmacotherapies in HF subgroups and different therapeutic strategies, are presented in Table 2. There were 70% of the patients with HFrEF having treatment with beta-blockers and RAASi compared to 52% among the patients with HFpEF.

**Table 2:** The registered pharmacotherapy and healthcare utilization during the one-year study period, in total and across the heart failure subgroups

	HFrEF	HFmrEF	HFpEF	HFndEF	Total	P-value
*Pharmacotherapy*						
RAASi, n (%)	379 (75)	346 (73)	474 (62)	608 (44)	1807 (58)	< 0.001^1^
BB, n (%)	425 (84)	395 (83)	534 (70)	800 (58)	2154 (69)	< 0.001^1^
MRA, n (%)	269 (53)	179 (38)	233 (31)	308 (22)	989 (32)	< 0.001^1^
Diuretics, n (%)	402 (79)	358 (76)	596 (78)	948 (68)	2304 (74)	< 0.001^1^
*Pharmacotherapy strategies*						
Only diuretics or no HF therapy	20 (4)	19 (4)	74 (10)	251 (18)	364 (12)	< 0.001^1^
BB or RAASi	105 (21)	109 (23)	252 (33)	573 (41)	1039 (33)	
BB and RAASi	357 (70)	319 (67)	394 (52)	516 (37)	1586 (51)	
BB, RAASi and MRA	25 (5)	27 (6)	44 (6)	49 (4)	145 (5)	
*Healthcare utilization*						
Hospital care						
*Inpatient care (IPC)*						
Hospital admissions, n (SD)	1.9 (1.4)	1.7 (1.5)	1.5 (1.6)	1.3 (1.3)	1.5 (1.4)	< 0.001^2^
Hospital days/IPC, n (SD)	14.4 (13.8)	11.9 (14.3)	11.0 (15.1)	8.8 (11.4)	10.7 (13.4)	< 0.001^2^
*Outpatient care (visits)*						
Physician, n (SD)	1.6 (2.2)	1.6 (2.1)	1.2 (1.8)	0.5 (1.3)	1.0 (1.8)	< 0.001^2^
Nurse, n (SD)	3.0 (6.1)	1.8 (3.9)	1.6 (8.1)	0.6 (4.8)	1.4 (5.9)	< 0.001^2^
Emergency department, n (SD)	0.2 (0.6)	0.2 (0.7)	0.2 (0.6)	0.2 (0.5)	0.2 (0.6)	< 0.001^2^
Primary care						
Physician (visits), n (SD)	5.0 (4.8)	6.3 (5.3)	6.2 (5.1)	4.8 (4.9)	5.4 (5.0)	< 0.001^2^
Nurse (visits), n (SD)	10.1 (11.9)	11.2 (13.2)	10.3 (13.2)	6.5 (10.1)	8.7 (11.9)	< 0.001^2^

*Note* HFrEF = heart failure with reduced ejection fraction, HFmrEF = heart failure with mildly reduced ejection fraction, HFpEF = heart failure with preserved ejection fraction, HFndEF = heart failure with no defined ejection fraction, n = numbers, RAASi = renin-angiotensin-aldosterone-system inhibition (includes Angiotensin-converting-enzyme inhibitors, Angiotensin receptor blockers and Angiotensin receptor neprilysin), BB = Betablocker, MRA = mineralocorticoid receptor antagonists, Diuretics = loop-diuretics, HF = heart failure, SD = standard deviation, IPC = Inpatient care

^1^ Pearson Chi-Square

^2^ One-way ANOVA

The extent of healthcare utilization for the different HF subgroups is displayed in Table 2. The highest number of hospital admissions, hospital inpatient care days and visits to physicians and nurses at hospital outpatient clinics was seen in HFrEF and HFmrEF patients.

Within 30 days, 364 patients (12%) had died and the corresponding number for one year was 873 (28%). Figure 1 displays the all-cause mortality, represented through Kaplan-Meier curves, both for the entire study cohort and separately for the different heart failure subgroups (HFrEF, HFmrEF, HFpEF, and HFndEF). Those patients with performed echocardiogram and specified HF phenotype had a one-year all-cause mortality rate of 19%.

An analysis using Spearman correlation between NT-proBNP and eGFR had a correlation of 29% (*p* < 0.001).

Within the entire cohort, 1941 (62%) consisted of individuals with one or less hospital admissions, while 1193 (38%) comprised those with two or more hospital admissions. Supplementary Table 3 in Appendix displays the distribution of the number of hospital admissions for the total cohort and its distribution among the HF subgroups. Table 3 displays a Cox regression model with the outcome of hospital admissions adjusted for HF-subgroups, kidney function, NT-proBNP levels, diabetes, COPD, recommended pharmacotherapy for HF (betablockers combined with RAASi) and visit to primary care physician.

**Table 3:** Cox regression with the outcome having at least one hospital admission during the study period of one year adjusted for HF-subgroups, kidney function and NT-proBNP levels

	HR	95.0% CI for HR		p-value
		Lower	Upper	
*HF-subgroups*				0.01
HFpEF	Reference			
HFmrEF	1.07	0.93	1.22	
HFrEF	1.10	0.97	1.26	
HF-ndEF	1.21	1.08	1.35	
*Kidney function*				< 0.001
> 60 ml/min	Reference			
30–60 ml/min	0.97	0.88	1.06	
< 30 ml/min	1.39	1.21	1.60	
*NT-proBNP levels*				< 0.001
HF unlikely	Reference			
Grey zone	1.23	1.02	1.49	
HF likely	1.71	1.42	2.06	
*Comorbidities*				
Cardiovascular disease	1.15	1.06	1.26	0.001
Diabetes	1.14	1.03	1.26	0.01
COPD	1.19	1.06	1.34	0.003
*Pharmacotherapy*				
BB and RAASi	0.80	0.73	0.88	< 0.001
*Healthcare utilization*				
Primary care physician	0.35	0.31	0.40	< 0.001

*Note* NT-proBNP = natriuretic terminal pro brain natriuretic peptide, HF = Heart failure, HR = Hazard ratio, CI = confidence interval, HFrEF = heart failure with reduced ejection fraction, HFmrEF = heart failure with mildly reduced ejection fraction, HFpEF = heart failure with preserved ejection fraction, HFndEF = heart failure with no defined ejection fraction, COPD = chronic obstructive pulmonary disease, BB = betablockers, RAASi = renin-angiotensin-aldosterone-system inhibition (includes Angiotensin-converting-enzyme inhibitors, Angiotensin receptor blockers and Angiotensin receptor neprilysin)

A univariate Cox regression analysis for one-year all-cause mortality for HF-subgroups, age, sex, comorbidities, renal function, NT-proBNP, pharmacotherapy (RAAS in combination with BB), hospital days and visits to primary care physician is shown in Table 4. Adjusted for age, sex, renal functional level, NT-proBNP level, comorbidities, and pharmacological treatment, the HFrEF and HFndEF groups had a significantly higher hazard ratio (HR) than the HFpEF group.

**Table 4:** Regression model for one-year all-cause mortality consisting of a Cox regression adjusted for Heart failure subgroups, confounding factors, exposure variables and healthcare utilization

	95% CI for hazard ratio (HR)			
	HR	Lower	Upper	P-value
*HF-phenotypes*				< 0.001
HFpEF	Reference			
HFmrEF	0.95	0.77	1.18	
HFrEF	1.12	0.91	1.38	
HFndEF	1.49	1.27	1.74	
*Gender*				
Women, n (%)^1^	0.90	0.80	1.02	0.10
Age (continous variable)	1.04	1.03	1.06	< 0.001
*Comorbidities*				
Hypertension	0.91	0.79	1.06	0.22
Cardiovascular disease	0.98	0.87	1.11	0.78
Atrial fibrillation, n (%)	1.06	0.93	1.20	0.38
Diabetes, n (%)	1.31	1.13	1.52	< 0.001
COPD, n (%)	1.51	1.29	1.77	< 0.001
*Kidney function*				< 0.001
> 60 ml/min, n (%)	Reference			
30–60 ml/min, n (%)	1.00	0.86	1.15	
< 30 ml/min, n (%)	1.88	1.56	2.28	
*NT-proBNP levels*				< 0.001
HF unlikely, n (%)	Reference			
Grey zone, n (%)	1.31	0.99	1.73	
HF likely, n (%)	2.09	1.59	2.76	
*Pharmacotherapy*				
Betablockers and RAASi	0.52	0.44	0.61	< 0.001
*Healthcare utilization*				
Inpatient care (days)	1.02	1.01	1.02	< 0.001
Hospital admissions	1.21	1.17	1.25	< 0.001
Outpatient care visits	0.96	0.95	0.98	< 0.001
Visit to primary care physician	0.16	0.14	0.19	< 0.001

*Note* CI = confidence interval, HR = hazard ratio, HF = Heart failure, HFpEF = heart failure with preserved ejection fraction, HFmrEF = heart failure with mildly reduced ejection fraction, HFrEF = heart failure with reduced ejection fraction, HFndEF = heart failure with no defined ejection fraction, n = numbers, COPD = chronic obstructive pulmonary disease, NT-proBNP = natriuretic terminal pro brain natriuretic peptide, RAASi = renin-angiotensin-aldosterone-system inhibition, MRA = mineralocorticoid receptor antagonists, Diuretics = loop-diuretics

## Discussion

In elderly patients with HF, healthcare utilization was generally high. Patients with HFndEF, renal impairment, elevated NT-proBNP levels, and concurrent cardiovascular disease (CVD), diabetes, and COPD were associated with an increased risk for hospitalization but lowered in patients having a primary care follow-up. The all-cause mortality rate was 28% within one year, most intense in the first 30-days in the HFndEF group but aligns after the initial 30-days between the HF subgroups. Factors such as age, impaired renal function, increased NT-proBNP, diabetes and COPD was associated with a higher risk of mortality but having treatment with beta-blockers and RAASi according to guidelines was associated with a lower risk for hospitalization and mortality.

In this current study, which encompasses patients newly diagnosed with HF monitored for one year, there was a notably elevated healthcare utilization compared to similar studies [10–13]. These comparative studies show an average hospital stay of approximately 6–7 days. However, present study reports an average hospital stay of 10.7 days. This discrepancy can be attributed to the inclusion of patients newly diagnosed with HF which would be a period when there is an enhanced need for hospital inpatient care. Additionally, patients with HFrEF had the highest hospitalization duration, averaging 14.4 days, further underlining the greater demand for hospital care within this specific patient subgroup. The patients in the present study made approximately 5–6 visits to a primary care physician, a result that aligns with findings from other studies [30]. Earlier studies have suggested that taking a proactive approach in primary care can effectively mitigate the requirement for hospitalization [29, 30]. Conducting a Cox regression analysis with hospital admissions as the outcome variable, the results indicated that a higher frequency of visits to primary care was associated with a reduced risk of hospital admissions. Within this study, the HF-subgroup did not exhibit any statistically significant influence on hospital admissions except for those patients with HFndEF. Conversely, the presence of kidney failure and elevated NT-proBNP values was associated with a heightened risk of hospital admission.

In 56% of the population an echocardiography was performed prior to HF diagnosis. A previous study investigating the same cohort as the present, but also including all patients with HF < 76 and ≥ 18 years of age, found that 57% had undergone an echocardiography examination [20]. This minimal difference between the entire population and the elderly, was somewhat surprising, since older patients would have been expected less likely to have undergone this examination. In the present study, the oldest patients (85–95 years old) had undergone an echocardiography examination in 46% of cases, compared to the patients aged 76 to 84 years, where the corresponding number was 64%. A previous Swedish study from 2009 investigating the adequacy of HF diagnosis found that 31% of patients had undergone an echocardiography examination and, in this perspective, there have been an improvement even among old adults [31]. Further, 8% of patients in the entire cohort had received a HF diagnosis based on clinical criteria, in the absence of both echocardiogram and NT-proBNP measurement. Electrocardiographic or chest x-ray abnormalities may have been present, but these are to be considered merely as supportive evidence of HF and are not classified as diagnostic criteria [20].

Recommended pharmacotherapies have generally been under prescribed and even more so when it comes to elderly patients [7, 10, 14, 31]. This may be partly attributed to healthcare providers assuming that comorbidities and advanced age imply frailty and a lower tolerance for recommended pharmacotherapy. However, it has previously been shown that the use of recommended pharmacotherapies for HFrEF was adequate also in older patients and similarly effective in the elderly patients as in the younger [18]. This study reveals that treatment with beta-blockers in combination with RAASi was associated with reduced mortality and hospitalization.

The high 30-day all-cause mortality observed in this study, amounted to 12% in the entire cohort and remarkably 18% in the HFndEF group. The one-year all-cause mortality rate was the highest in the HFndEF and the HFrEF group, as would have been expected. Naturally, higher age was correlated to a higher mortality risk, as were elevated levels of NT-proBNP. Impaired renal function, COPD and diabetes was also associated with an increased mortality risk.

## Limitations

This study comprises patients diagnosed with HF at the onset, and the proportion of patients with a diagnostic echocardiography has been collected. Among those who had echocardiograms, it was possible to establish the HF-phenotype, making this aspect unique for a population-based study. However, it cannot be guaranteed whether the echocardiography took place during the acute phase or during the recovery phase. There might be patients having an echocardiography at a different hospital outside RH, which were not accessible for this research. These cases are considered rare and would not affect the result. In a population-based cohort study, a certain percentage of patients diagnosed with HF have not been assessed using echocardiography. This naturally presents a limitation when conducting analyses across the entire cohort.

The pharmacotherapy data is based on picked up medications, which enhances accuracy. However, it should be noted that the specific dosage of these drugs could not be ascertained in this study. The pharmacotherapy reported in the current study aligns with the recommendations available at the time of the study period. Subsequent guidelines have introduced changes, particularly regarding SGLT-2 inhibition, which have not been considered in this context [7, 14].

In this study, the chosen threshold values assumed that patients initially presented with acute deterioration, resulting in a new HF diagnosis. However, it cannot be assured that every patient experienced an acute deterioration at the time of diagnosis. The prevailing Swedish guidelines for newly diagnosed heart failure were applied [24, 32]. It could be postulated that kidney dysfunction may influence elevated NT-proBNP levels, yet this influence is evaluated as constrained, given the Spearman correlation of 29%.

In the current study, heart failure phenotypes were defined using previous EF criteria, identifying HFrEF as < 40%, HFmrEF as 40–50%, and HFpEF as greater > 50% [33]. Recent guidelines have updated these definitions to HFrEF as < 40%, HFmrEF as 41–49%, and HFpEF as > 50% [7]. Since the echocardiographic evaluations were performed according to the older standards, the assessment concluded that it would be most appropriate to apply the guidelines that were established at the time of the examination.

As this study was a retrospective observational study, it is not possible to establish causal inference or determine coefficients for both inter- and intra-observer variability. Accounting for all potential confounding variables in this retrospective study can be challenging, thereby impacting the ability to establish causality. Accurately establishing the timeline of events may also prove difficult, as it relies on the reliability of recordings. Additionally, pharmacotherapy practices have evolved over time according to guidelines, rendering today’s guidelines inapplicable to the study period. In addition to the degree of COPD and diabetes, future studies could assess HF patients’ possible degree of anemia, possible iron deficiency and the treatment of this, in relation to hospitalization and mortality. It would also be of interest to evaluate the prescribed dosage of the different recommended pharmacotherapies and its association with hospitalization and mortality.

## Conclusions

This study with elderly patients with newly diagnosed heart failure shows that renal impairment, elevated NT-proBNP values and CVD, diabetes and COPD were associated with increased risk of mortality and hospital admission within one year. Treatment with beta-blockers in combination with RAASi was associated with reduced mortality and hospital admission even at an advanced age. Follow-up with a visit to a primary care physician was associated with reduced risk for hospital admissions. This suggests that monitoring and follow-up, also in primary care, may be important for the prognosis.

### Electronic supplementary material

Below is the link to the electronic supplementary material.

Supplementary Material 1

Supplementary Material 2

## Data Availability

The datasets produced and scrutinized in the present study are not accessible to the public, in compliance with the provisions of the Swedish Health and Medical Services Act concerning the Secrecy Act. However, these datasets may be obtainable from Region Halland upon a reasonable request directed to the corresponding author. Access will be subject to a specific review conducted by the Regional Consultative Committee for data collection in Region Halland.

## Abbreviations

BB: betablockers
CI: confidence interval
COPD: chronic obstructive pulmonary disease
CVD: cardiovascular disease
Diuretics: loop-diuretics
EF: ejection fraction
EGFR: estimated glomerular filtration rate
HF: heart failure
HFrEF: heart failure with reduced ejection fraction
HFmrEF: heart failure with mildly reduced ejection fraction
HFpEF: heart failure with preserved ejection fraction
HFndEF: heart failure with no defined ejection fraction
HR: hazard ratio
IPC: inpatient care
MRA: mineralocorticoid receptor antagonists
n: number
NT-proBNP: natriuretic terminal pro brain natriuretic peptide
RAASi: renin-angiotensin-aldosterone-system inhibition (includes angiotensin-converting-enzyme inhibitors, angiotensin receptor blockers and angiotensin receptor neprilysin)
RH: Region Halland
RHIP: Regional Healthcare Information Platform
SD: standard deviation
QoL: quality of life
