# Supplementary material for: Factors affecting hospitalization and mortality in a retrospective study of elderly patients with heart failure
Source: BMC Cardiovasc Disord. 2024 Apr 26;24:227. doi: 10.1186/s12872-024-03871-6 (PMC11046923; doi:10.1186/s12872-024-03871-6)
Supplement: Supplementary file 1 — Supplementary Material 1 [file 12872_2024_3871_MOESM1_ESM.docx]

**Appendix**

Appendix-Table 1. ICD-10 codes relating to the comorbid conditions examined in the current study.

| **Disease** | **ICD-10 codes** |
| --- | --- |
| Heart failure | I110, I420, I423 – I432, I438, I500 – I501, I509 |
| Hypertension | I10 - I15 |
| Ischemic heart disease | I20 - I25 |
| Cerebrovascular insult | I60 – I69 |
| Atrial fibrillation | I48 |
| Diabetes mellitus | E10 – E14 |
| Chronic obstructive pulmonary disease | J44 |

Appendix-Table 2. The evidence-supported medications recommended for managing heart failure with reduced left ventricular function, as outlined in the study.

| **Medication (ATC-code)** |
| --- |
| *Angiotensin-converting-enzyme inhibitors (C09)* |
| Captopril |
| Enalapril |
| Lisinopril |
| Ramipril |
| Trandolapril |
| *Betablockers (C07)* |
| Bisoprolol |
| Carvedilol |
| Metoprolol |
| Nebivolol |
| *Angiotensin receptor blockers (C09)* |
| Candesartan |
| Valsartan |
| Losartan |
| *Mineralocorticoid receptor antagonists (C03DA)* |
| Eplerenone |
| Spironolactone |
| *Angiotensin receptor neprilysin inhibitors (C09DX04)* |
| Sacubitril/Valsartan |
| *Sodium glucose cotransporter-2 antagonists* |
| dapagliflozin |
| empagliflozin |

Appendix Table 3. The distribution of the number of hospital admissions for the total cohort and its distribution among the HF subgroups.

| **Hospital admissions (n)** | **HFrEF,**  **n (%)** | **HFmrEF, n (%)** | **HFpEF,**  **n (%)** | **HFndEF, n (%)** | **Total,**  **n (%)** |
| --- | --- | --- | --- | --- | --- |
| 0 | 47 (9) | 81 (17) | 199 (26) | 381 (27) | 708 (27) |
| 1 | 206 (41) | 172 (36) | 281 (37) | 574 (41) | 1233 (39) |
| 2 | 125 (25) | 120 (25) | 135 (18) | 250 (18) | 630 (20) |
| 3 | 58 (11) | 52 (11) | 67 (9) | 99 (7) | 276 (9) |
| 4 | 45 (9) | 25 (5) | 47 (6) | 41 (3) | 158 (5) |
| 5 | 13 (3) | 18 (4) | 17 (2) | 20 (1) | 68 (2) |
| >5 | 13 (3) | 6 (1) | 18 (2) | 24 (2) | 61 (2) |

Note: HF= Heart failure, HFrEF=heart failure with reduced ejection fraction, HFmrEF= heart failure with mildly reduced ejection fraction, HFpEF= heart failure with preserved ejection fraction, HFndEF= heart failure with no defined ejection fraction, n=number.
