# Supplementary material for: Factors affecting hospitalization and mortality in a retrospective study of elderly patients with heart failure
Source: BMC Cardiovasc Disord. 2024 Apr 26;24:227. doi: 10.1186/s12872-024-03871-6 (PMC11046923; doi:10.1186/s12872-024-03871-6)
Supplement: Supplementary file 2 — Supplementary Material 2 [file 12872_2024_3871_MOESM2_ESM.docx]

**Appendix – Figure**

Appendix Figure 1. Illustrates a flow diagram depicting the progression of the entire research study.


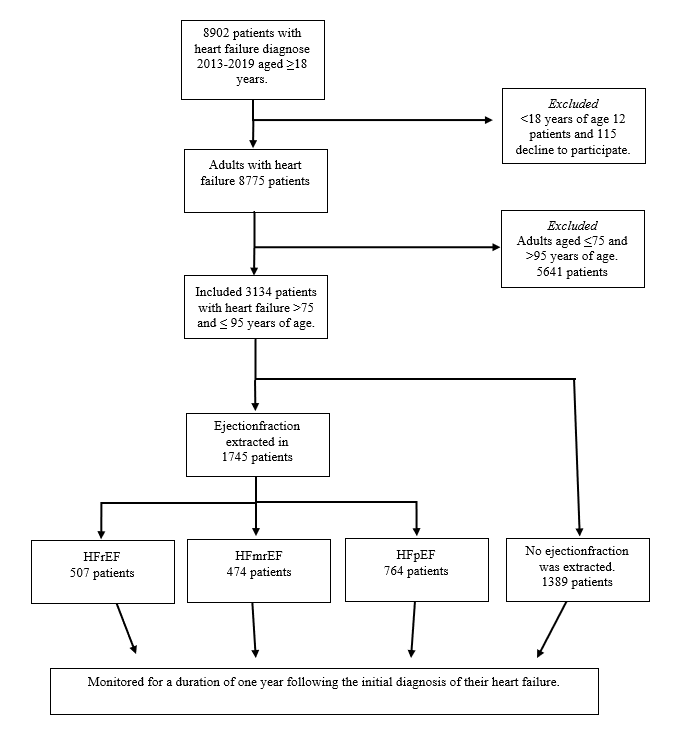


Note: HFrEF=heart failure with reduced ejection fraction, HFmrEF= heart failure with mildly reduced ejection fraction, HFpEF= heart failure with preserved ejection fraction.
